# Protective effect of antioxidants on the pre-maturation aging of mouse oocytes

**DOI:** 10.1038/s41598-017-01609-3

**Published:** 2017-05-03

**Authors:** Li-Feng Liang, Shu-Tao Qi, Ye-Xing Xian, Lin Huang, Xiao-Fang Sun, Wei-Hua Wang

**Affiliations:** 1 Key Laboratory of Major Obstetrics Diseases of Guangdong Province, the Third Hospital Affiliated of Guangzhou Medical University, Guangdong, China; 2 New Houston Health, Houston, Texas United States of America

## Abstract

Pre-maturation aging of immature oocytes may adversely affect the fate of an oocyte. Oxidative stress is one of the most detrimental factors affecting oocyte developmental competence and maturation during aging. In this study, experiments were designed to examine whether supplementation of antioxidants in a culture medium could protect immature mouse oocytes from damages caused by oxidative stress. Mouse oocytes at germinal vesicle stage were prevented from meiosis resumption and cultured in a medium with or without antioxidants for 12–36 h to allow oocytes to undergo aging. After aging, oocytes were cultured for maturation. Nuclear maturation, mitochondria activity, spindle morphology and DNA integrity were examined after maturation. It was found that antioxidants had protective effects on the oocytes in terms of nuclear maturation, functional mitochondria, spindle morphology and DNA integrity. As aging time was prolonged from 12 to 36 h, the protective effect of antioxidants became more obvious. However, as compared with oocytes without aging, it was found that aging significantly inhibited nuclear maturation, impaired mitochondria function, and damaged the spindle and DNA. These results indicate that pre-maturation aging is detrimental to oocytes’ competence to undergo maturation and other cellular activities, and antioxidants can protect oocytes from damages caused by aging.

## Introduction

Assisted reproductive technology, especially *in vitro* fertilization (IVF), has been widely used for treatment of infertility in humans. However, transferrable embryos and embryo implantation rates are still low^1, 2^. A healthy oocyte is important for successful fertilization, embryo development and subsequent implantation after transfer^3^. As oocyte growth in different follicles and/or ovaries does not occur simultaneously, some oocytes in fast growing follicles may be aging at the germinal vesicle (GV) stage, through a process we designated as “pre-maturation aging”, before oocyte maturation triggering by gonadotropins. Pre-maturation aging may adversely affect the outcome of maturation^4^.

Oxidative stress (OS) is considered to be a prominent mediator associated with oocyte aging and causes poor embryonic development^5, 6^. Quantity and quality of oocytes are greatly decreased because of apoptosis induced by OS^7^. It is believed that the balance between reactive oxygen species (ROS) and antioxidants within the oocytes is critical to cell functions, such as chromosome segregation^8, 9^, mitochondria activity^10, 11^, spindle assembly^12^, ATP level maintenance^13^ and DNA methylation^14^. Therefore, ROS may affect oocyte growth during follicle growth *in vivo*. Oocyte development to GV stage, meiotic resumption and fertilization are time sensitive events, and oocyte aging at any of these stages may affect their competence to undergo maturation, fertilization and/or development to an embryo or fetus^15, 16^. However, during ovarian stimulation for multiple oocyte growth and collection, 10–14 days are necessary for oocytes to become fully grown at GV stage. During this period, some follicles grow faster than others. In order to allow slowly growing follicles to reach the appropriate size so that a fully grown oocyte can be obtained, fast growing oocytes have to wait until most follicles reach a size suitable for triggering to induce meiosis resumption. During this waiting period (up to 36–72 hours), the oocytes in the fast growing follicles may undergo aging. This is frequently observed in patients with advanced maternal age^17^. Although the exact mechanism(s) for poor embryo development from these patients are unknown, it is possible that oocytes in fast growing follicles become aged before maturation triggering due to unsynchronized follicle growth. If excessive ROS are produced by mitochondria during oocyte aging and there are insufficient antioxidants to scavenge them, the equilibrium of ROS and antioxidants will be disrupted^18^, and apoptosis is accelerated^19^. These may be some of the major causes of the poor oocyte quality in patients who were stimulated for longer periods of time.

3-isobutyl-1-methylxanthine (IBMX) can be used as a nonspecific phosphodiesterase inhibitor for modulating cyclic adenosine monophosphate (cAMP) levels to prevent GV-stage oocytes from meiosis resumption during pre-maturation^20^. cAMP plays a critical role in maintaining the meiotic arrest of mammalian oocytes and in inducing their maturation^21^. Therefore, a temporary inhibition of meiosis in oocytes can be achieved by treatment with IBMX, resulting in oocytes arresting at GV stage. Supplementation of IBMX in a culture medium can maintain the oocytes at GV stage and can be used to mimic *in vivo* human ovarian stimulation before maturation triggering. It has been found that treatment of immature oocytes with IBMX can reduce hydrogen peroxide via increased gap-junctional communication to improve oocyte developmental competence and subsequent embryo development^22^.

Antioxidant supplementation has been proven to protect oocytes against ROS and OS^23^. Metal ions can be accumulated through food, water or air during a lifetime. It has also been found that accumulation of heavy metal ions is a major factor in inducing excessive ROS production within cells, and additional amounts of antioxidants are required to scavenge the excessive ROS. Sodium citrate, α-lipoic acid (ALA) and acetyl-L-carnitine (ALCAR) are antioxidants that play important roles in protecting mammalian cells against oxidative stress by scavenging free radicals^24, 25^. Treatment of oocytes with ALCAR during *in vitro* maturation (IVM) can improve the oocyte quality by increasing the proportion of mature oocytes with even mitochondrial distribution^26^. Meanwhile, supplementation of ALA to culture media improved *in vitro* development of follicles, mitochondrial activity, gene expression and embryo development in older female mice^23, 27^.

However, almost all studies on oocyte aging were focused on oocytes after meiosis I^28, 29^. As of our knowledge, no study so far has reported such effects on immature oocyte aging, i.e. pre-maturation aging. The pre-maturation aging of oocytes at GV stage is a common phenomenon in human IVF when oocytes are not triggered for maturation at an appropriate time. Therefore, in the present study, mouse oocytes were maintained at GV stage in a medium supplemented with IBMX and antioxidants for 12–36 h, and then processed to IVM to investigate the effects of antioxidants (sodium citrate, ALA and ALCAR) on nuclear maturation and other cellular functions, such as mitochondria activity, meiotic spindle formation, chromosome configuration and DNA integrity.

## Results

### Antioxidants improved oocyte maturation after pre-maturation aging

**Figure 1:**
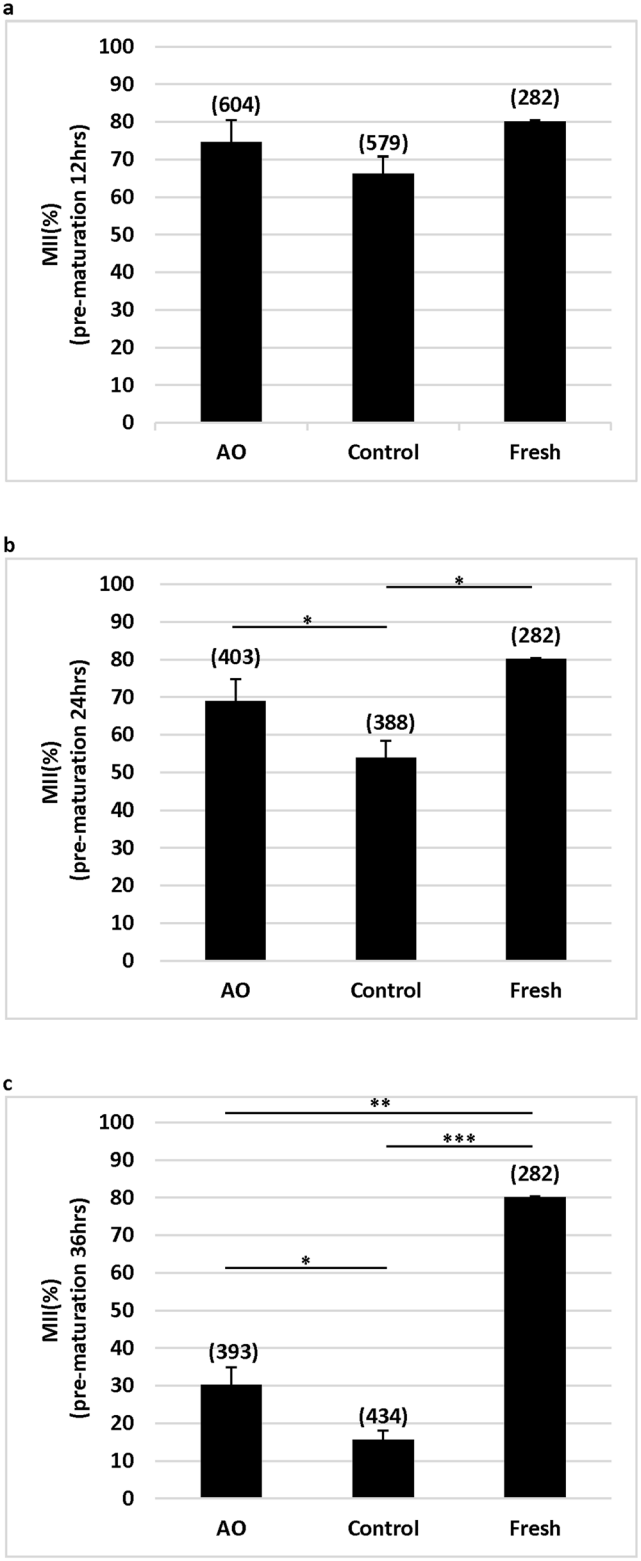
Effects of antioxidants on oocyte maturation after pre-maturation aging and IVM. Oocytes at GV stage were directly cultured (Fresh) or pre-maturation aging for 12 (**a**) 24 (**b**) and 36 h (**c**) in a medium supplemented with (AO) or without (control) antioxidants before IVM. *P < 0.05; **P < 0.01; ***P < 0.001. No. of oocytes at each group was shown in the bracket. AO: antioxidants.

To investigate the effects of antioxidants on the competence of oocytes that matured to metaphase II (MII) stage, oocytes were examined after pre-maturation aging for 12, 24 and 36 h, and then underwent IVM for 14 h. Culture media were either supplemented with antioxidants, or were not supplemented at all. Partial GV oocytes were directly processed to IVM for 14 h without pre-maturation aging (fresh oocytes). As shown in Fig. 1a, when oocytes were cultured for pre-maturation aging for 12 h and then for IVM, the proportions of oocytes reached MII stage were no statistical differences among the groups. However, when the pre-maturation aging time was prolonged to 24 h, the proportion of oocytes reached MII stage were significantly decreased (P < 0.05) in the control group without antioxidants (53.92%) as compared with the fresh group (80.14%) and the antioxidant group (68.91%) (Fig. 1b). When the pre-maturation aging time was prolonged to 36 h, the proportions of oocytes reached MII stage reduced significantly (P < 0.01 at least) in all aging groups as compared with the fresh group (80.14%), although more (P < 0.05) oocytes reached MII stage in the group with antioxidants (30.23%) as compared with the group without antioxidants (15.66%) (Fig. 1c).

### Effects of antioxidants on mitochondria distribution in pre-maturation aging oocytes

**Figure 2:**
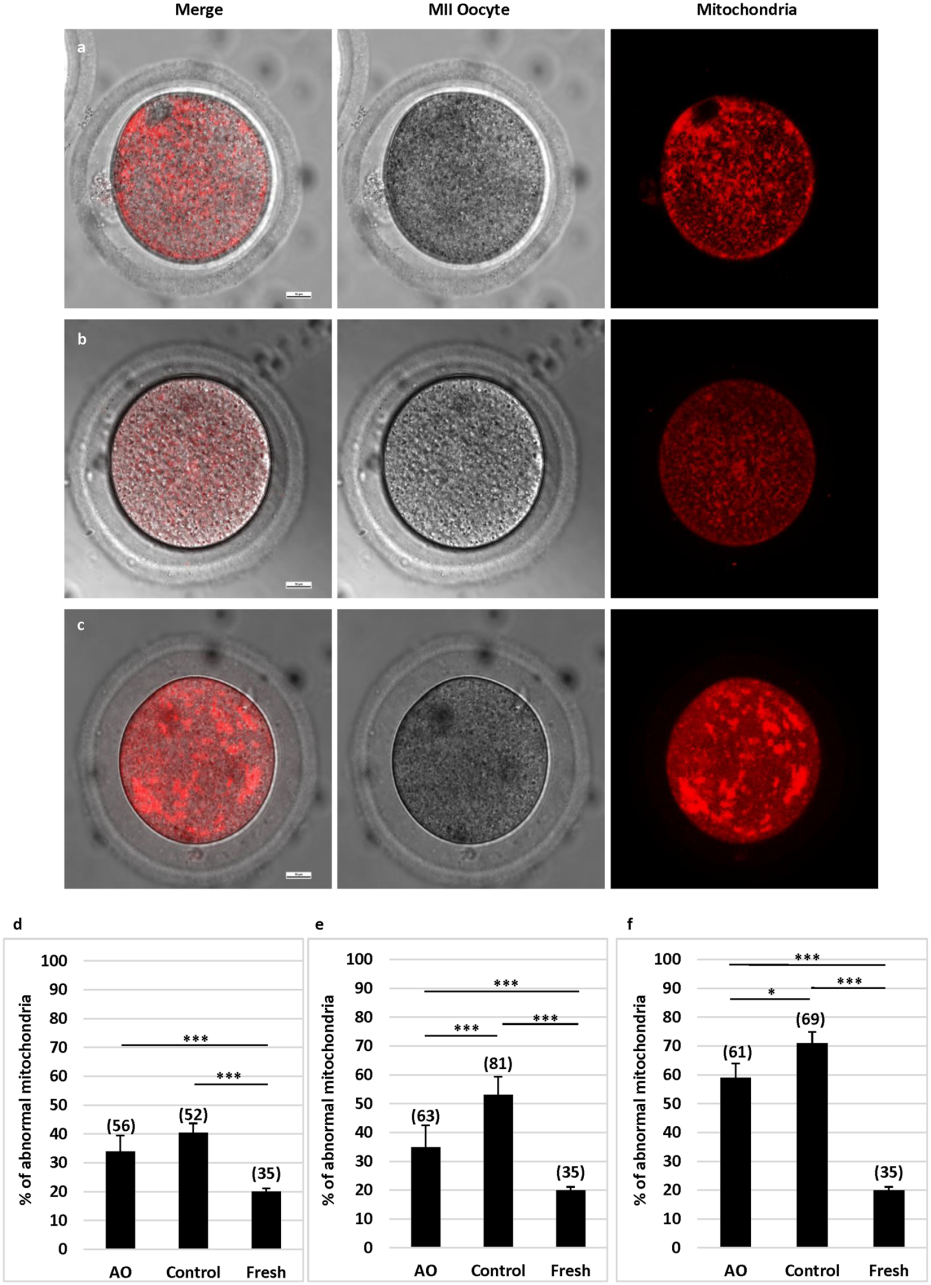
Effects of antioxidants on mitochondrial distribution in oocytes at MII stage after pre-maturation aging and IVM. Mitochondrial distribution patterns in oocytes were detected by fluorescence microscopy using MitoTracker Red. (**a**) Shows polarized distribution. (**b**) Shows homogeneous distribution. Both a and b were considered as normal mitochondria distribution patterns. (**c**) Shows large heterogeneous clump distribution, which was considered as abnormal mitochondria distribution pattern. Bar = 10 µm. (**d**) Shows proportions of oocytes with abnormal mitochondria distribution after 12 h pre-maturation aging and IVM. (**e**) Shows proportions of oocytes with abnormal mitochondria distribution after 24 h pre-maturation aging and IVM. (**f**) Shows proportions of oocytes with abnormal mitochondria distribution after 36 h pre-maturation aging and IVM. *P < 0.05; **P < 0.01; ***P < 0.001. No. of oocytes at each group was shown in the bracket. AO: antioxidants.

Mitochondrial function is an important prerequisite for oocyte maturation and subsequent fertilization and development. Accordingly, we used a mitochondria tracker to examine active mitochondria in the oocytes after IVM. There are three kinds of active mitochondria distribution patterns observed in mouse eggs. Normal mitochondria distribution is either polarized distribution (Fig. 2a), or homogeneous distribution (Fig. 2b), while abnormal mitochondria distribution is large, heterogeneous clump distribution (Fig. 2c). As shown in Fig. 2d–f, we found that pre-maturation aging significantly (P < 0.001) increased the number of oocytes (33.93–71.01%) with abnormal mitochondria distribution, as compared with fresh oocytes (20%), in a time-dependent manner. However, the proportions of oocytes with abnormal mitochondria distribution were reduced when oocytes were cultured in the medium with antioxidants as compared with oocytes cultured without antioxidants in all groups. However, significant (P < 0.05 at least) differences were observed only in the 24 and 36 h groups.

### Antioxidants improved normal spindle formation and chromosome configuration during oocyte aging

**Figure 3:**
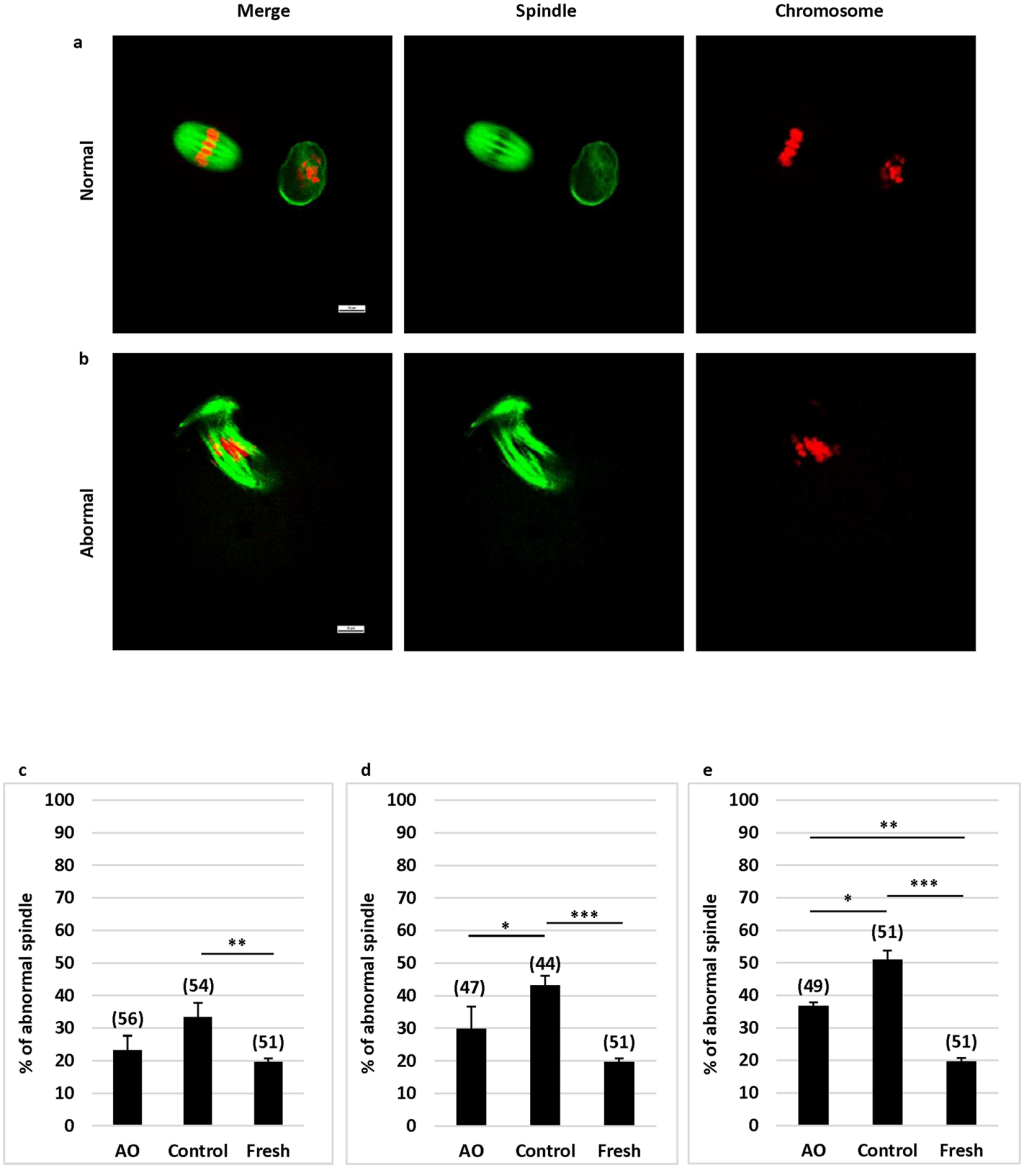
Effects of antioxidants on spindle morphology and chromosome alignment in oocytes at MII stage after pre-maturation aging and IVM. (**a**) Shows an oocyte with a normal spindle and chromosome alignment. (**b**) Shows an oocyte with an abnormal spindle and chromosome alignment. Green: a-Tubulin; Red: chromosomes. (Bar = 10 µm). (**c**) Shows percentages of oocytes with abnormal spindle and/or misaligned chromosomes in oocytes after 12 h pre-maturation aging and IVM. (**d**) Shows percentages of oocytes with abnormal spindle and/or misaligned chromosomes in oocytes after 24 h pre-maturation aging and IVM. (**e**) Shows percentages of oocytes with abnormal spindle and/or misaligned chromosomes in oocytes after 36 h pre-maturation aging and IVM. *P < 0.05; **P < 0.01; ***P < 0.001. No. of oocytes at each group was shown in the bracket. AO: antioxidants.

Oocyte quality was also determined by spindle morphology and chromosome alignment. A normal oocyte has a spindle with a typical barrel-shape and regularly aligned chromosomes on the spindle equatorial plane (Fig. 3a), while an abnormal oocyte has a spindle with irregular microtubule distribution and presence of chromosomes that do not align on the spindle equatorial plane (Fig. 3b). Oocytes with abnormal spindle and/or chromosome alignment may affect chromosome segregation and result in aneuploidy after fertilization^30^.

As shown in Fig. 3c,d, the proportions of oocytes (33.33–50.98%) with an abnormal spindle and/or chromosome misalignment were significantly (P < 0.01 at least) higher in aging oocytes cultured in the medium without antioxidants as compared with fresh oocytes (19.61%) in all groups (12–36 h). If antioxidants were supplemented in the medium during 12 and 24 h of oocyte aging (Fig. 3c and d), the proportions of oocytes with an abnormal spindle and/or chromosome alignment were similar to those in fresh oocytes, and a significant (P < 0.01) difference was observed if pre-maturation aging was extended to 36 h (36.73% vs 19.61%) (Fig. 3e). Supplementation of antioxidants significantly (P < 0.05) reduced the number of oocytes with abnormal spindle formation in 24 and 36 h of aging groups as compared with the control (without antioxidant supplement).

### Antioxidants prevented DNA damage in oocytes during aging

**Figure 4:**
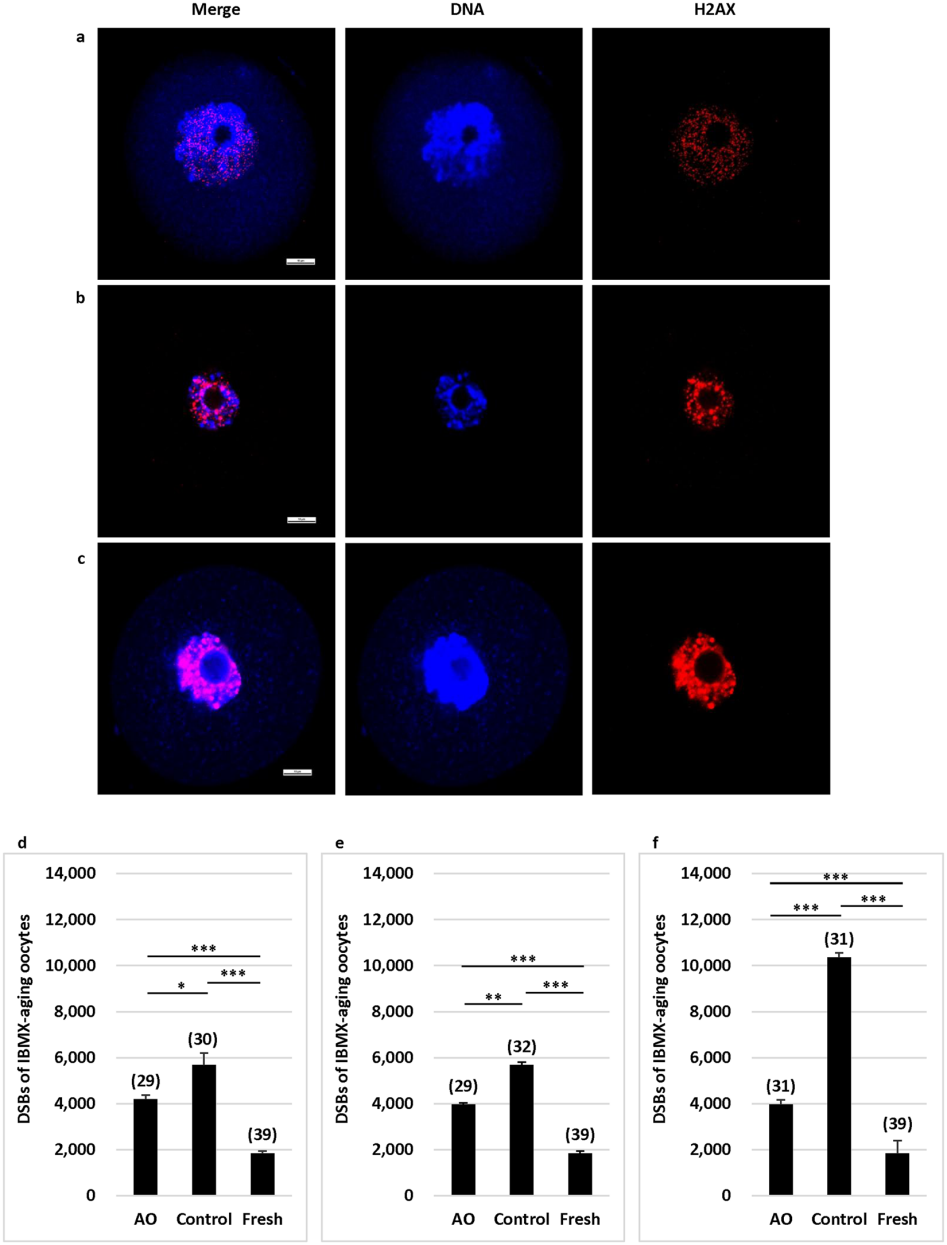
Effects of antioxidants on DNA integrity in the oocytes at GV stage after pre-maturation aging and IVM. Fluorescence images show DNA and γ-H2AX in oocytes at GV stage after pre-maturation aging and IVM. (**a**) Shows an oocyte with slight DNA damage. (**b**) Shows an oocyte with moderate DNA damage. (**c**) Shows an oocyte with severe DNA damage. Red: γ-H2AX; Blue: DNA. (Bar = 10 mm). (**d**) Shows levels of fluorescence intensity of γ-H2AX in oocytes after 12 h pre-maturation aging and IVM. (**e**) Shows levels of fluorescence intensity of γ-H2AX in oocytes after 24 h pre-maturation aging and IVM. (**f**) Shows levels of fluorescence intensity of γ-H2AX in oocytes after 36 h pre-maturation aging and IVM. *P < 0.05; **P < 0.01; ***P < 0.001. No. of oocytes at each group was shown in the bracket. AO: antioxidants.

Imbalances between ROS and antioxidants, or excessive ROS can lead to different forms of DNA damage. However, DNA double-strand breaks (DSBs) are particularly hazardous to the cell because they can lead to genome rearrangement, threatening oocytes’ normal activities and even survival^31^. Therefore, we used DSBs marker γ-H2AX to examine the fluorescence intensity in the oocytes that arrested at GV stage. As shown in Fig. 4a–c, various degrees of fluorescence staining, which indicate different DNA damages, can be observed in the oocytes.

We found that mean intensity of γ-H2AX fluorescence in fresh oocytes was low (Fig. 4d–f). As the pre-maturation aging was processed to 12–36 h, fluorescence intensities in aging oocytes were significantly (P < 0.01 at least) increased in a culture time dependent manner. Again, although higher levels of γ-H2AX fluorescence intensities were observed in all pre-maturation aging groups as compared with those in fresh eggs, antioxidants in the culture medium significantly (P < 0.05 at least) reduced the levels of γ-H2AX fluorescence intensities in all groups as compared with those without antioxidants. These results indicated that antioxidants could protect oocytes from ROS-induced DNA damage.

## Discussion

It has been reported that oocyte aging at MII stage causes poor fertilization and embryo development, and antioxidant supplementation to culture medium can increase the developmental potential of oocytes or embryos^25, 32^, but no study has examined oocyte aging at GV stage or the effects of antioxidants on pre-maturation aging. Our study, as of our knowledge, for the first time, showed that aging at GV stage is detrimental to oocyte nuclear and cytoplasmic maturation. Some major cellular functions, such as mitochondria function, meiotic spindle formation/chromosome configuration, and DNA integrity, are affected during pre-maturation aging. If antioxidants are supplemented in the culture medium, normal function of oocytes can be protected significantly.

During human ovarian stimulation for IVF, oocytes in different sizes of follicles develop asynchronously, which in turn causes some oocytes to grow faster than others. The oocytes in fast growing follicles have to wait for a few days until most follicles reach suitable sizes for oocyte maturation triggering. This will cause pre-maturation aging of oocytes at GV stage, and the degree of aging depends on the waiting time before maturation triggering. This occurs in most patients, but more commonly in poorly responding women and/or advanced maternal aged women^33^. A reduction of the quantity and quality of oocytes is the major characteristic in these women. Usually, fewer and/or poor oocytes are collected for IVF in these patients, which eventually leads to poor outcomes in subsequent embryo development^34^. It is believed that oocyte aging occurs after meiosis resumption if oocytes are not fertilized in time; however, here we provided evidence that aging of oocytes at GV stage exists, and it may be more common than oocyte aging after maturation. This kind of aging may be caused by many factors, but cumulative production of ROS inside a follicle and/or within oocytes may be one of them. The present study indicates that antioxidants can protect oocytes from these damages by maintaining cellular activities.

During IVM, oocytes are usually cultured under conditions with high concentration of oxygen, resulting in oocyte aging by the increased production of ROS^35, 36^. Increased generation of ROS overwhelms the antioxidants’ capacity, leading to OS to harm oocytes. These are the reasons that oocytes are much more susceptible to OS during IVM than *in vivo* maturation. It has been reported that antioxidants, such as ALA, ALCAR, melatonin or Vitamin E, have significant anti-aging effects for oocytes or embryos under *in vitro* culture systems^25, 37, 38^. It has also been reported that metals, such as iron, copper, nickel or cadmium, are present in culture media and in the human body, and are confirmed sources of free radical production^39^. Although we do not know whether the ROS within aging oocytes is from metals or other sources, our results obtained in the present study indicate that sodium citrate, ALA and ALCAR are able to scavenge these ROS, thus oocyte maturation, spindle, mitochondria and DNA damages within oocytes could be reduced significantly.

Aging associated changes in mitochondrial distribution and formation of large heterogeneous mitochondria may lead to insufficient energy supplies in the oocytes, a possible cause for arrest of oocyte meiosis^40^. In our study, the proportion of oocytes with abnormal mitochondria distribution can be significantly reduced in the medium supplemented with antioxidants than in the medium without antioxidants, indicating that sodium citrate, ALA, and ALCAR have a protective anti-aging effect by maintaining functional mitochondria. Functional mitochondria is important for sufficient ATP production within oocytes. A several of cellular events, such as the assembly of meiotic spindle and chromosome segregation, are energy-dependent^41^, shortage of ATP would cause disturbances in spindle formation and function, which in turn would result in maturation arrest and/or aneuploidy formation^42^.

In the present study, the proportion of oocytes with abnormal spindle morphology was lower in the oocytes cultured in the medium with antioxidants than without antioxidants. This result was consistence with a previous study in which oocytes from aged mouse also had lower incidence of abnormal spindle morphology and chromosome misalignment if oocytes were cultured in a medium supplemented with antioxidants^23^. These results suggest that antioxidant supplement in the culture medium is important for normal spindle formation/separation and chromosome segregation, especially in aging oocytes.

Once DNA damage occurs, DNA repair proteins can sense DNA DSBs and transmit signals to checkpoint proteins to arrest the cell cycle^43^. A significantly lower level of DSBs was observed in fresh oocytes than in aging oocytes, indicating that aging causes DNA damage. A previous study showed that DNA damage did not inhibit or delay GV breakdown in porcine oocytes, but inhibited the final oocyte maturation^44^. In our study, most aging oocytes also underwent GV breakdown (data not shown), but more oocytes arrested at metaphase I stage when the pre-maturation aging time was prolonged. To examine whether sodium citrate, ALA, and ALCAR can protect oocytes from DNA damage caused by ROS, we used a DNA DSBs marker γ-H2AX to evaluate the DNA damage on arrested oocytes and our data indicate that these antioxidants can reduce DNA damage during pre-maturation aging. Again, this protective effect may be resulted from scavenging of ROS by antioxidants.

In conclusion, our results indicate that antioxidants have beneficial effects on oocyte maturation after pre-maturation aging by forming a normal spindle and maintaining a functional mitochondria, while diminishing the DSBs. These protective effects by antioxidants can overcome oxidative stress by scavenging ROS before they can damage cells. However, aging of oocytes at GV stage is a complicated cellular event, and supplementation of culture media with antioxidants alone does not improve oocyte quality significantly, although it is better than a lack of antioxidant supplement. Furthermore, although it is not practical, double egg collections in poorly stimulated IVF patients, along with IVM of oocytes from fast growing follicles may be able to produce more viable embryos, and culture media supplemented with antioxidants may benefit oocyte quality during egg manipulations and culture.

## Materials and Methods

### Animals and ethic statement

7–8 week-old female ICR mice were used for the experiments. Mouse care and use were conducted in strict accordance with the recommendations in the Guide for the Care and Use of Laboratory Animals of the National Institutes of Health. The animal protocol was approved by the Animal Care and Use Committee of the Third Hospital Affiliated of Guangzhou Medical University. Mice were housed and fed under 12:12 h light:dark cycle before immature oocytes were retrieved.

### Oocyte collection

Female ICR mice were treated with 5 IU pregnant mare serum gonadotropin (PMSG) by i.p. injections. Mice were then killed by cervical dislocation and ovaries were collected 48 h after PMSG injection. After washing in α-MEM culture medium (Corning, Manassas, VA, USA) supplemented with 10% fetal bovine serum (FBS) (Corning, Manassas, VA, USA), ovaries were excised and oocytes at GV stage were collected under a stereomicroscope (200×).

### Experimental design and *in vitro* culture of oocytes

Oocytes at GV stage were divided into three groups: (1) Antioxidants group: oocytes were exposed to pre-maturation aging in M16/M2 medium (Sigma) containing 0.5 mM IBMX (Sigma, St. Louis, MO, USA) and 10 μmol sodium citrate (Sigma), 25 μmol ALA (Sigma) and 20 μmol ALCAR (Sigma); (2) Control group: oocytes were exposed to pre-maturation aging in M16/M2 medium with 0.5 mM IBMX but without any antioxidant; (3) Fresh group: oocytes were cultured in M16/M2 media for IVM without aging. For pre-maturation aging, oocytes in antioxidant group and control group were cultured in 20 μl of droplets at 37 °C in a humidified atmosphere of 5.0% CO_2_ for 12, 24, and 36 h, respectively. After pre-maturation aging, oocytes were cultured without IBMX for 14 h in M2 medium supplemented with 10% FBS, recombinant 75 mIU/mL follicle-stimulating hormone (Sigma), 0.5 IU/mL human chorionic gonadotropin (Sigma), and 5 ng/mL recombinant epidermal growth factor (Sigma). Oocytes in fresh group were directly cultured for 14 h in the IVM medium without pre-maturation aging. After IVM, oocytes with extrusion of the first polar body were considered as MII stage. Oocytes at MII stage were counted and then used for assessment of mitochondrial distribution and spindle morphology. The oocytes arrested at GV stage after IVM were used for examination of DNA DSBs.

### Immunofluorescence and confocal microscopic examination of spindles and DSBs

The protocols for examination of spindles and DSBs were essentially the same as previous studies^42–45^. Briefly, for spindle and chromosome examination, oocytes were fixed with 4% paraformaldehyde in phosphate-buffered saline (PBS) for 30 min at room temperature. After being permeabilized with 0.5% Triton X-100 for 30 min, oocytes were blocked in a blocking solution (PBS containing 1% bovine serum albumin) for 60 min, and then incubated with 1:200 mouse anti-α-tubulin-FITC antibody (Sigma) for 2 h. After oocytes were washed in a washing solution (PBS with 0.1% Tween 20 and 0.01% Triton X-100) for three times, 5 min each, oocytes were stained for chromosomes by 10 µg/ml propidium iodide (Sigma) for 15 min, and then were mounted on glass slides.

For DNA DSBs examination, oocytes at GV stage were fixed and blocked with the same methods mentioned above. After blocking treatment, oocytes were cultured with 1:100 rabbit anti-γ-H2AX (Cell Signaling Technology, Danvers, MA, USA) in the blocking solution overnight, and then labeled with 1:500 donkey anti-rabbit IgG secondary antibody, Alexa Fluor 546 conjugate (Invitrogen, Carlsbad, CA, USA) for 1.5 h at room temperature. Oocytes were then stained for DNA examination with 10 µg/ml 4′,6-diamidino-2-phenylindole dihydrochloride (Sigma) for 15 min and finally were mounted on glass slides.

Spindle morphology and DSBs γ-H2AX were observed under a laser-scanning confocal microscope (Nikon, Tokyo, Japan) equipped with argon and helium–neon lasers (excitation wavelength: 488 and 543 nm, respectively). The fluorescence intensity of γ-H2AX was analyzed by ImageJ.

### Assessment of mitochondrial distribution

To assess the mitochondrial distribution in oocytes, oocytes at MII stage were incubated in M2 medium containing 12.5 μM Mitotracker Red CMXRos (Life technologies, Eugene, Oregon, USA) in the dark at 37 °C for 30 min. After incubation, oocytes were washed three times (10 min each) in M2 medium and then were observed under a confocal microscope immediately (Nikon, Tokyo, Japan).

### Statistical analysis

All experiments were repeated at least three times. The initial numbers of oocytes were 40–60 for each group. After IVM, oocytes at MII stage were used for examination of spindles and mitochondria, and oocytes at GV stages were used for γ-H2AX examination. For these subsequent experiments, each 10–20 oocytes were assigned to each group. All data were expressed as percentages and then mean ± SEM was obtained from experimental replications. Statistical analyses were performed using SPSS version 18 (SPSS Inc., Chicago, IL, USA) and chi-square test or student *t*-test was used to compare the differences between groups. Difference at P < 0.05 was considered to be statistically significant.
